# Editorial: Genomics and gene editing of orphan plants

**DOI:** 10.3389/fpls.2023.1277625

**Published:** 2023-09-13

**Authors:** Ali Movahedi, Boas Pucker, Saeid Kadkhodaei

**Affiliations:** ^1^ College of Biology and the Environment, Nanjing Forestry University, Nanjing, China; ^2^ Institute of Plant Biology, TU Braunschweig, Braunschweig, Germany; ^3^ Agricultural Biotechnology Research Institute of Iran, Agricultural Research, Education and Extension Organization, Isfahan, Iran

**Keywords:** CRISPR-Cas, orphan crops, genomics, gene editing, genetic improvement

Orphan crops are of vital importance for global food security. These crops have the capacity to provide nutritious sustenance and show strong adaptability to changing climates. The cultivation and conservation of these crops contribute to safeguarding biodiversity and supporting small-scale farmers by offering diverse income opportunities. These plants display durability to survive under challenging environmental conditions such as drought, extreme temperatures, and pest exposure. Scientists can enhance crop quality and productivity by utilizing controlled breeding methods and advanced genetic engineering techniques like the CRISPR/Cas system (**Figure 1**). Moreover, gene editing tools like TALENs and ZFNs have been employed to tackle the unique difficulties, such as diseases, nutrition, breeding, etc., posed by orphan crops (Venezia and Creasey Krainer, 2021). Fragrant Rice is known for its smell because of its defective *badh2* allele encoding betaine aldehyde dehydrogenase (BADH2), which produces 2-acetyl-1-pyrroline (2AP). TALENs were engineered to disrupt the *OsBADH2* gene, increasing the 2AP content through homozygous mutations. Therefore, targeted mutagenesis using TALENs is useful in creating important agronomic traits (Shan et al., 2015). Genomics and gene editing technologies accelerate breeding by enabling scientists to identify and select genes underlying desired traits quickly (Chen et al., 2019), while efficient transformation processes are crucial for applying Cas9 to target sequences, enhancing the accuracy and precision of gene editing techniques (Cattivelli et al., 2008; Movahedi et al., 2023). Plants with specific traits are developed by minimizing off-target effects and ensuring successful Cas9 conjugation with a guide RNA molecule, which helps Cas9 locate and bind to the corresponding DNA sequence. The deployment of this technology has dramatically accelerated breeding programs by swiftly incorporating helpful traits into plant varieties (Wolter et al., 2019). *Phyllostachys edulis* holds significant global importance as a monopodial bamboo species. The lack of a genetic transformation system poses challenges in elucidating the functionalities of genes that govern crucial traits and conducting molecular breeding in Moso bamboo. Researchers have achieved a 5% increase in plant regeneration and transformation efficiency using plant growth regulators and antibiotic screening. CRISPR/Cas genetic changes have been used to enhance desirable traits in Moso bamboo, such as pest resistance (Huang et al.). Researchers can improve crop productivity, plant quality, yield, and nutritional content by targeting specific genes with CRISPR/Cas (Curtin et al., 2018). This technology offers several advantages for orphan crops, including transferring valuable traits such as disease and stress resistance (Sedeek et al., 2019). Successful application of the CRISPR/Cas system has been demonstrated in sesame, where cytochrome *P450* genes involved in sesamin and sesamolin biosynthesis were successfully targeted, resulting in expected genetic modifications (You et al.). Sequencing the genomes of different orphan plant varieties provides the basis for identifying essential genes responsible for desirable traits (Gasparini et al., 2021). Genomic insights into orphan plants offer opportunities to manipulate genes to enhance traits like taste, aroma, and nutritional value. For instance, in *Physalis pruinosa*, genomic resources and efficient transformation techniques were developed (Lemmon et al., 2018). Through CRISPR/Cas genome editing, orthologues of tomato domestication and improved genes were mutated, significantly enhancing plant architecture, flower production, and fruit size. This rapid development of targeted allelic diversity and novel breeding germplasm showcases the potential for improving plants distantly related to crops (Lemmon et al., 2018). By specifically targeting genes involved in secondary metabolite synthesis, CRISPR/Cas editing has the potential to enhance the nutritional value of orphan plants by improving flavor profiles and increasing the production of beneficial compounds. In one study, Cas9 was employed to inactivate the kauniolide synthase genes in *Cichorium intybus*, interrupting sesquiterpene lactone biosynthesis. These findings highlight the potential of CRISPR/Cas in enhancing the nutritional value of chicory taproots by altering the biosynthesis of sesquiterpene lactones (Cankar et al.). Moreover, genomics and gene editing technologies, like CRISPR/Cas, advance agriculture by editing plant genomes, enhancing sustainability, and reducing reliance on chemical pesticides and fertilizers (Qaim, 2020). However, it is crucial to address the regulatory and ethical considerations surrounding gene editing in orphan crops (Sprink et al., 2016). Public engagement and science communication are necessary to fully realize the potential of genomics and gene editing and promote responsible and sustainable agricultural practices. Gene knock-in using CRISPR-Cas in plants is significant, as it enables the insertion of favorite genes into specific locations within the plant genome (Zhang et al., 2017; Movahedi et al., 2022). Site-specific gene knock-in using CRISPR/Cas enables precise and targeted insertion of desired genes into specific loci within the plant genome, making it highly significant in plant biology and crop enhancement. This technique has been applied to *Chlamydomonas reinhardtii* to facilitate the insertion of an optimized foreign bacterial phytase gene at a specific site (Shahabadi et al.). These findings indicate that CRISPR/Cas can be used to insert a foreign gene into the nucleus, showing its effectiveness in accurately adding genes to specific locations without causing adverse effects on gene expression.

**Figure 1:**
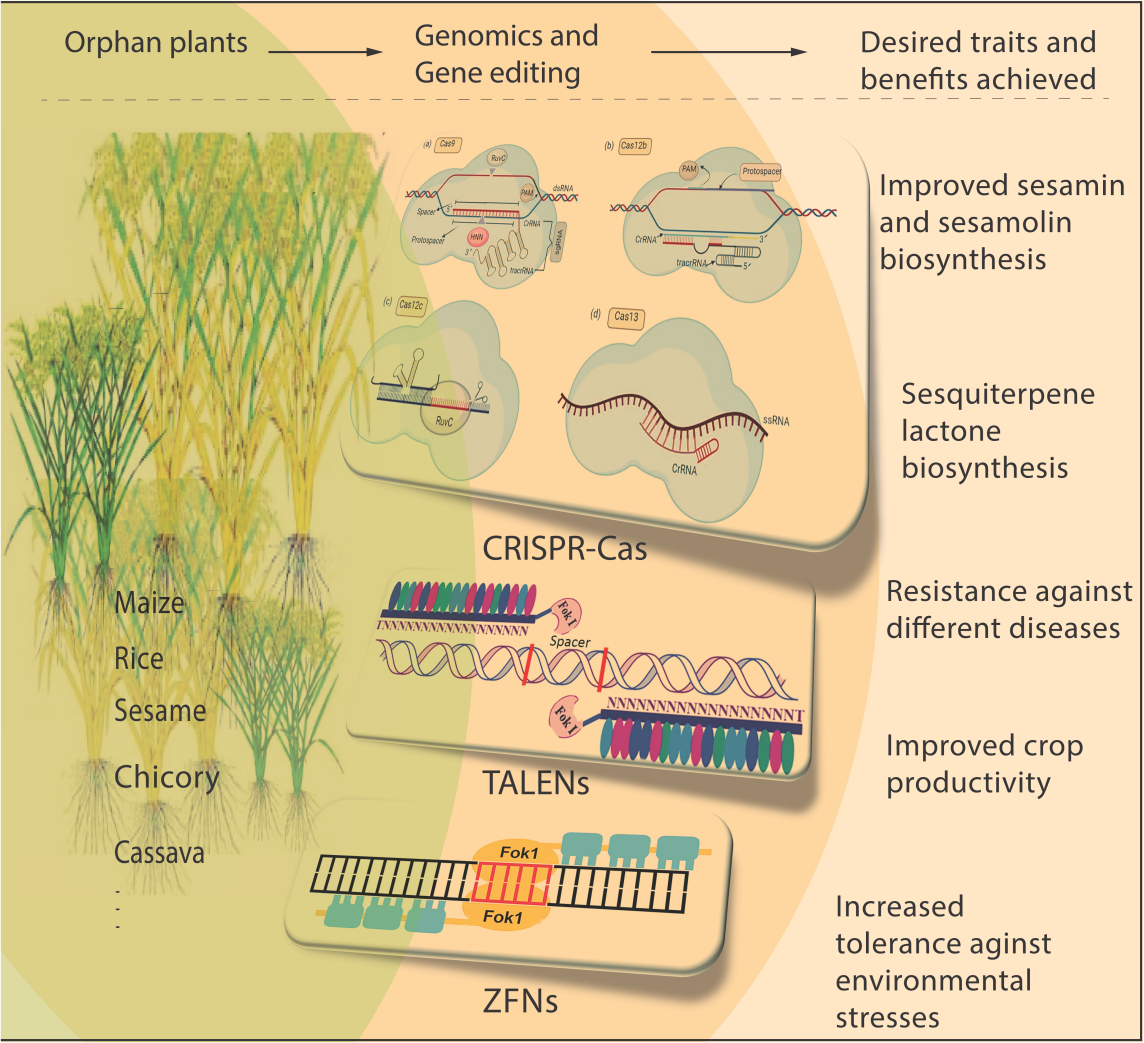
Pictorial diagram illustrating genomics and gene editing technologies for improving desirable traits in orphan crops. Genome editing technologies are being developed to enhance the productivity and disease resistance of orphan crops like rice, maize, cassava chicory, etc. For instance, sesame can benefit from sesamin and sesamolin biosynthesis, while chicory can benefit from sesquiterpene lactone biosynthesis. The CRISPR/Cas, TALENs, and ZFN technologies have transformed the field of plant genetics by providing precise tools to modify specific traits by altering the genome editing of plants.

To summarize, combining genomics and gene editing offers excellent opportunities to tackle worldwide concerns regarding food security. It also provides a means to enhance the flavor, fragrance, and nutritional content of underutilized plants. These technologies facilitate the engineering of improved crop varieties with beneficial characteristics, leading to better nutrition and overall health.

## Author contributions

AM: Conceptualization, Project administration, Supervision, Writing – original draft, Writing – review & editing. BP: Validation, Writing – review & editing. SK: Validation, Writing – review & editing.
